# Activation and Migration of Human Skeletal Muscle Stem Cells In Vitro Differently Rely on Calcium Signals

**DOI:** 10.3390/cells11101689

**Published:** 2022-05-19

**Authors:** Axel Tollance, Stéphane Koenig, Nicolas Liaudet, Maud Frieden

**Affiliations:** 1 Department of Cell Physiology and Metabolism, University of Geneva Medical Center, 1211 Geneva, Switzerland; axel.tollance@unige.ch; 2 Bioimaging Core Facility, University of Geneva Medical Center, 1211 Geneva, Switzerland; nicolas.liaudet@unige.ch

**Keywords:** live cell imaging, single-cell analysis, reserve cells, serum-induced activation, calcium signals, migration

## Abstract

Muscle regeneration is essential for proper muscle homeostasis and relies primarily on muscle stem cells (MuSC). MuSC are maintained quiescent in their niche and can be activated following muscle injury. Using an in vitro model of primary human quiescent MuSC (called reserve cells, RC), we analyzed their Ca^2+^ response following their activation by fetal calf serum and assessed the role of Ca^2+^ in the processes of RC activation and migration. The results showed that RC displayed a high response heterogeneity in a cell-dependent manner following serum stimulation. Most of these responses relied on inositol 1,4,5-trisphosphate (IP_3_)-dependent Ca^2+^ release associated with Ca^2+^ influx, partly due to store-operated calcium entry. Our study further found that blocking the IP_3_ production, Ca^2+^ influx, or both did not prevent the activation of RC. Intra- or extracellular Ca^2+^ chelation did not impede RC activation. However, their migration potential depended on Ca^2+^ responses displayed upon stimulation, and Ca^2+^ blockers inhibited their movement. We conclude that the two major steps of muscle regeneration, namely the activation and migration of MuSC, differently rely on Ca^2+^ signals.

## 1. Introduction

Skeletal muscle regeneration occurs throughout the life of a human being. It requires the presence of muscle stem cells (MuSC), formerly called satellite cells, which are nonproliferating cells maintained in a quiescent state and able to be activated, meaning they can re-enter the cell cycle in response to specific stimuli. MuSC activation occurs, for example, after muscle fiber injury [1] and is triggered by a diversity of inflammatory molecules released by immune and vascular cells [2]. Upon activation, MuSC proliferate as myoblasts with two possible fates. While the majority fuses and regenerates the damaged fibers, a small part replenishes the pool of MuSC for further regeneration [3,4]. MuSC activation is thus the initial step of skeletal muscle regeneration, but this event is very difficult to study. Indeed, the manipulations required to isolate MuSC are sufficient to trigger their activation [5,6,7], making the study of the initial events of MuSC activation challenging. However, it is possible to obtain in vitro MuSC from human myoblasts [8,9,10]. After myoblast differentiation, around 70% of cells differentiate and fuse as myotubes, which are large polynucleated cells [8], while the remaining 30% stop their cell cycle in the G_0_ phase to enter a quiescent state and acquire stem cell characteristics [8,9]. These cells are called reserve cells (RC) and are the in vitro counterparts of MuSC [11]. RC activation can then be induced, for instance, with fetal calf serum, which allows the signaling pathways involved in the first steps of this process to be followed [11,12].

Ca^2+^ signaling is essential for myotube differentiation [13,14,15], muscle regeneration [16], and the maintenance of the MuSC pool [10]. In particular, store-operated Ca^2+^ entry (SOCE) was shown to be essential for muscle differentiation [13,14,17,18]. This pathway relies on the activation of the plasma membrane Ca^2+^ selective Orai channels by the endoplasmic/sarcoplasmic reticulum (ER/SR)-localized STIM proteins, which are activated upon Ca^2+^ store depletion [19]. In addition to myotube formation, previous studies have reported the involvement of Ca^2+^ signals in MuSC activation. Both Ca^2+^ release from the stores and Ca^2+^ influx have been described as sources of cytosolic Ca^2+^ elevation depending on the type of stimulation, namely release from the ER/SR through ryanodine receptors in amphibian larvae [16], influx through Ca^2+^ channels linked to mechanical stress [20], or transient receptor potential canonical (TRPC)-linked influx in response to FGF2 stimuli [21] in murine models. However, detailed analysis of the Ca^2+^ response occurring in MuSC upon activation and its possible link with the fate of these cells is missing, and almost nothing is known about the role of Ca^2+^ signals in RC in humans.

In this work, we used an in vitro model of human quiescent RC to study the first steps of their activation. This model allows cell activation to be precisely distinguished from subsequent cell proliferation. We first analyzed the Ca^2+^ response of RC after serum stimulation and uncovered a great heterogeneity of Ca^2+^ signals, which relied on Ca^2+^ release and, to a variable extent, on Ca^2+^ entry. We then established whether the Ca^2+^ responses were causally linked to the fate of RC in terms of activation and migration. We found that their re-entry into the cell cycle occurred independently of any Ca^2+^ response, while the pattern of Ca^2+^ signals affected their migration during the 48 h following serum stimulation.

## 2. Materials and Methods

### 2.1. Cell Culture

Human primary myoblasts were isolated from semitendinosus muscle samples obtained after orthopedic surgery (surgical waste) on patients without known muscular diseases. All samples were collected anonymously after obtaining written consent and approval from the University of Geneva (protocol CCER no. PB_2016-01793 (12-259) and was accepted by the Swiss Regulatory Health Authorities and approved by the “Commission Cantonale d’Ethique de la Recherche” from the Geneva Cantonal Authorities, Switzerland). The purification of myoblasts was performed as previously described [9]. Briefly, muscles were mechanically and enzymatically (trypsin-EDTA, 25200056 Thermo Fisher, St. Louis, MO, USA) dissociated to keep only mononucleated cells, followed by cell amplification and sorting by FACS (CD56+; CD82+ and CD146+) to purify myoblasts. Myoblasts were cultured in a growth medium (GM) containing 15% of fetal calf serum (FCS) until reaching confluency, after which the differentiation was triggered by replacing GM with differentiation medium (DM), a medium without FCS (composition of GM and DM has been previously described [22]). The cultures of RC and myotubes were used after 48 h in DM.

### 2.2. Immunofluorescence and Activation Assay

Cells were fixed in PBS with 4% paraformaldehyde, permeabilized, and blocked in PBS containing 0.3% of Triton X-100 and 5% goat serum. Immunofluorescence was performed by overnight incubation at 4 °C with the following primary antibodies: mouse anti-α-actinin (1:500, A7811, Sigma, Taufkirchen, Germany) and rabbit anti-MEF2c (1:500, 5030S, Cell Signaling, Danvers, MA, USA). Secondary antibodies were incubated for 75 min at room temperature (RT) with Alexa Fluor^®^ 488-conjugated goat antimouse IgG (1:1000, A11029, Life Technologies, Carlsbad, CA, USA) and Alexa Fluor^®^ 546-conjugated goat antirabbit IgG (1:1000, A11030, Life Technologies, Carlsbad, CA, USA).

RC activation and myoblast proliferation were determined by EdU assay (Click-iT™ Plus EdU Cell Proliferation Kit for Imaging, Alexa Fluor™ 647 dye, C10640, ThermoFisher, Carlsbad, CA, USA) based on the provider’s protocol. Nuclei were stained using ProLong^®^ Gold Antifade Reagent with DAPI (ref. P36931, Life Technologies, Carlsbad, CA, USA). Images were acquired with a widefield AxioImager M2 microscope (ZEISS, Germany) through a 20× objective (EC Plan-Apochromat 20×/0.8).

To assess effect of Ca^2+^ on activation and/or proliferation, cells were stimulated with GM supplemented by chemicals including the phospholipase C inhibitor U73122 (5 µM, U6756-5MG, Sigma, Taufkirchen, Germany) or its inactivated control U73343 (5 µM, U6881-1MG, Sigma, Taufkirchen, Germany), the G-protein-coupled receptor αq inhibitor YM254890 (10 µM, AG-CN2-0509-MC05, AdipoGen, Liestal, Switzerland), the inhibitor of Orai1 and -2 channels GSK7975a (10 µM, AOB4124, AOBIOUS, Gloucester, MA, USA), EGTA (0.3 and 0.6 mM), Gd^3+^ (50 µM), and BAPTA-AM (10 µM, B6769, Thermofisher, Carlsbad, CA, USA). In the same way, cells were stimulated with DM supplemented with endothelin-1 (100 nM, Cat. No. 1160, Tocris, Abingdon, UK). To estimate the proportion of activated and nonactivated RCs, we quantified the proportion of DAPI^+^/MEF2c^−^/EdU^+^ cells compared to the DAPI^+^/MEF2c^−^ cell population. Analysis of the immunofluorescence images was performed using ImageJ software.

### 2.3. Calcium Measurements

Cells were loaded with Fura-2-AM (2 µM, F1201, ThermoFisher, Carlsbad, CA, USA) at room temperature and in the dark for 30 min in a medium containing 135 mM NaCl, 5 mM KCl, 1 mM MgCl_2_, 10 mM HEPES, 2 mM CaCl_2_, and 10 mM glucose, with pH adjusted at 7.45 with NaOH (Ca^2+^ medium). Following the incubation, cells were washed and kept for 10 min in the same medium to allow de-esterification of the dye. Calcium-free medium (CF medium) was composed of 135 mM NaCl, 5 mM KCl, 1 mM MgCl_2_, 10 mM HEPES, 10 mM glucose, and EGTA 1 mM, with pH adjusted at 7.45 with NaOH.

Ratiometric images of Ca^2+^ signals were recorded using a ZEISS Axio Observer A1 microscope equipped with a Lambda XL illumination system (Sutter Instrument, Novato, CA, USA), with the excitation wavelengths switched between 340 nm (ET340x; Chroma, Bellows Falls, VT, USA) and 380 nm (ET380x; Chroma, Bellows Falls, VT, USA). Emission was collected through a 415 DRLP dichroic mirror and a 510WB40 filter (Omega Optical, Brattleboro, VT, USA) by a cooled 16-bit CMOS camera (pco.Edge sCMOS, Visitron Systems, Puchheim, Germany). Image acquisition was performed with the VisiWiew software (Visitron Systems, Puchheim, Germany), and the analysis was done with Fiji and MATLAB software. Cells were recorded for 30 min after stimulation, with a ratio taken every 2 s. RC were stimulated with CA medium containing 15% serum, and cells were treated with the same chemicals described previously. Stimulations with endothelin-1 (100 nM, Cat. No. 1160, Tocris, Abingdon, UK) and ATP (100 µM, Sigma, Taufkirchen, Germany) were performed in CA medium.

### 2.4. Timelapse and Movement Tracking

To reduce UV phototoxicity, cytosolic Ca^2+^ was recorded using Cal520-AM (5 µM, AAT Bioquest^®^, Sunnyvale, CA, USA; excitation at 480 nm), and nuclei were labeled with SPY650-DNA (1 nM, 1 h SC501 Spyrochrome^®^, Stein am Rhein, Switzerland; excitation at 630 nm) to follow cell movement and allow their individual identification. After incubation in the dark at 37 °C in DM for 80 min, the cells were washed and kept for 20 min in DM to allow de-esterification of the Cal520-AM dye. Cells were stimulated with GM, and cytosolic Ca^2+^ concentration was measured for 10 min at a 20× magnification with a picture taken every 15 s. After this initial Ca^2+^ recording, pictures of the phase contrast and SPY650 of the same field were captured at a magnification of 10× for 48 h with one picture taken every 15 min. Experiments were performed on a ZEISS Axio Observer Z1 with definite focus 2, thermoregulated at 37 °C, and CO_2_ controlled. Timelapse recordings of the Ca^2+^ signals and tracking experiments were automatically registered using phase correlation after upsampling of the last frame of the Ca^2+^ acquisition to reach the same lateral resolution as the one used for the tracking experiment. This allowed the identification and classification of the tracked cells according to their Ca^2+^ response category. The tracking was performed automatically with Imaris 9.7.2 (Bitplane, Zürich, Switzerland) using the spot module under systematic user supervision and with a manual correction to minimize cell position errors. Thereafter, the tracking information (cell positions and time) was processed with MATLAB R2021a (The Mathworks, Bern, Switzerland) to compute different features, such as the maximum speed and the mean displacement (average distance between two consecutive timepoints).

### 2.5. Statistics

Data are presented as mean ± SEM, and statistical differences were determined using the test specified in the legend of each figure, where * *p* < 0.05, ** *p* < 0.01, ***p < 0.001, and **** *p* < 0.0001. For comparison of two populations, T-test or Mann–Whitney test were used depending on the normality of the data set. For comparison of more than two populations, ANOVA or Kruskal–Wallis test were used depending on the normality of the data set.

## 3. Results

### 3.1. Reserve Cells Display Heterogeneous Calcium Responses upon Serum Stimulation

**Figure 1:**
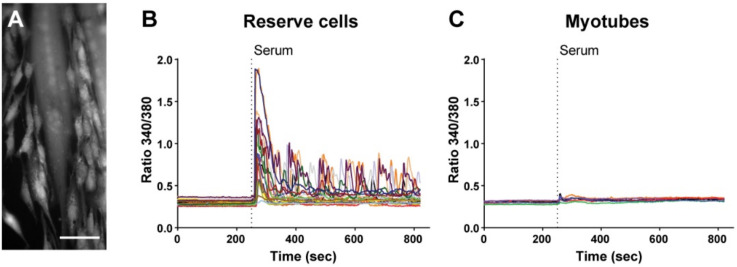
Serum induces Ca^2+^ responses in reserve cells but not in myotubes. (**A**) Representative image of Fura-2-loaded cells 48 h after differentiation. The multinucleated myotubes and the mononucleated RC can be easily recognized. Scale bar: 100 µm. (**B**,**C**) Cytosolic Ca^2+^ recordings showing the serum-induced cytosolic Ca^2+^ response in RC (**B**) but not in myotubes (**C**). Each panel (**B**,**C**) is the response of a representative coverslip of *n* = 21 reserve cells and *n* = 6 myotubes acquired in three independent experiments.

In vitro human primary myoblast differentiation leads to the formation of two different cell types, namely the postmitotic multinucleated myotubes and the quiescent mononucleated RC [10]. After 48 h in differentiation medium, we stimulated the cells with a Ca^2+^ medium supplemented with 15% fetal calf serum (serum stimulation) and analyzed the Ca^2+^ response for 30 min. Serum stimulation triggered a strong Ca^2+^ response in RC, while no response was observed in myotubes (Figure 1). These results show that, as expected, myotubes and RC do not share the same ability to respond to activating stimuli. However, myotubes displayed Ca^2+^ elevation when stimulated with ATP, showing that the absence of serum-induced response did not reflect any kind of cell damage (Figure S1).

**Figure 2:**
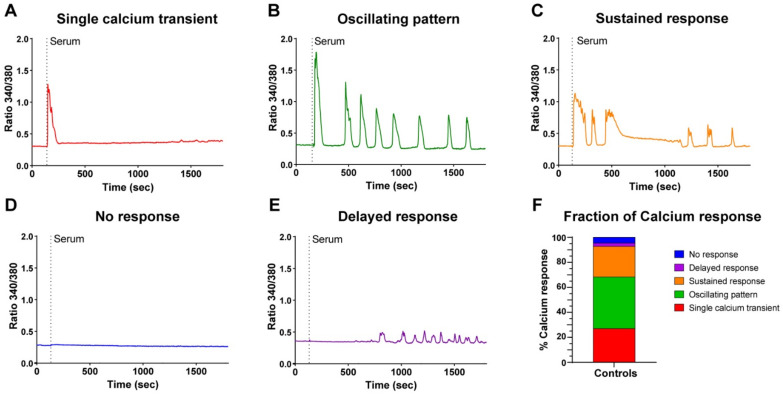
Heterogeneity of the serum-induced Ca^2+^ responses of reserve cells. Myoblasts were differentiated for 48 h to obtain myotubes and RC. The cells were loaded with Fura-2 and stimulated by 15% serum. Representative examples of the five different patterns of responses (**A**–**E**). (**F**) Percentage of each type of response; 673 cells were analyzed from 15 independent experiments.

A careful analysis of the responses revealed that RC displayed different patterns of Ca^2+^ signals, which we classified into five categories: a single Ca^2+^ transient (Figure 2A), a Ca^2+^ transient followed by an oscillating pattern (Figure 2B), a Ca^2+^ transient followed by a phase containing sustained Ca^2+^ elevation (Figure 2C), no response at all (Figure 2D), or a delayed response without the initial Ca^2+^ transient in around 5% of cells (Figure 2E). Overall, 92% of the RC presented a clear Ca^2+^ elevation, corresponding to the first three categories (Figure 2F). These different patterns of responses were consistently observed despite the primary cells coming from different donors (seven different donors in these experiments). Hence, these results highlight that the RC population is highly heterogeneous in its Ca^2+^ response to serum stimulation.

### 3.2. Calcium Release and Calcium Influx Are Used by Reserve Cells to Generate Calcium Signals

**Figure 3:**
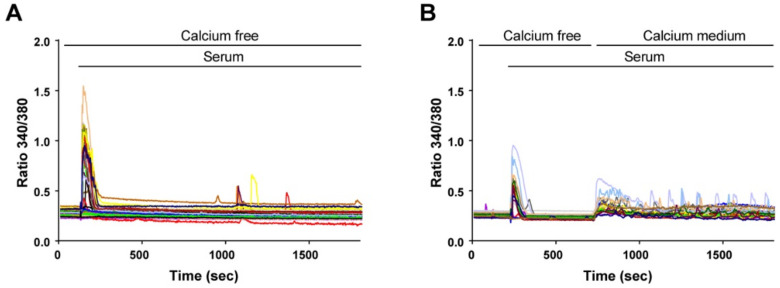
Extracellular Ca^2+^ is required to sustain the oscillatory responses. Myoblasts were differentiated for 48 h to obtain myotubes and RC. The cells were loaded with Fura-2 and stimulated by 15% serum. (**A**) In a Ca^2+^-free medium, the Ca^2+^ response was only transient. (**B**) Ca^2+^ oscillations resumed when Ca^2+^ was readded in the extracellular medium. Each panel is the representative response of *n* = 41 reserve cells (**A**) and *n* = 21 reserve cells (**B**) acquired in two independent coverslips.

To determine the source of cytosolic Ca^2+^ elevation, we first stimulated the cells in the absence of extracellular Ca^2+^. As expected, only one Ca^2+^ transient was observed under this condition (Figure 3A), showing that subsequent Ca^2+^ elevations depended on Ca^2+^ entry. Indeed, readding extracellular Ca^2+^ after 8 min resumed the Ca^2+^ signal (Figure 3B).

**Figure 4:**
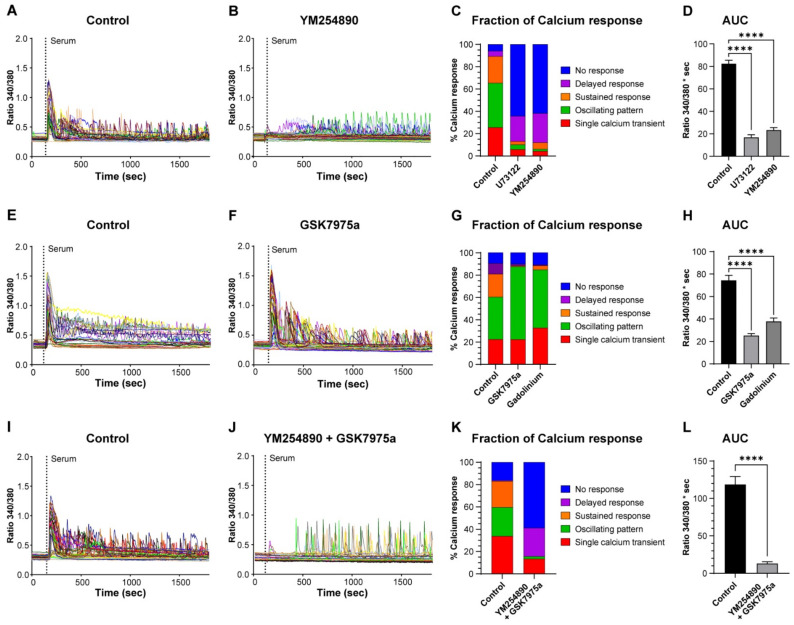
IP_3_-induced Ca^2+^ release and Ca^2+^ entry account for most of the serum-induced Ca^2+^ response. Myoblasts were differentiated for 48 h to obtain myotubes and RC. The cells were loaded with Fura-2 and stimulated by 15% serum in the control condition (**A**,**E**,**I**) and after preincubation with 10 µM YM254890 (**B**), 10 µM GSK7975a (**F**), or both inhibitors (**J**). Each panel (**A**,**B**,**E**,**F**,**I**,**J**) is the response of a representative coverslip, with the control of each inhibitory condition displayed. The proportion of each type of Ca^2+^ response is shown after inhibition of IP_3_ production with YM254890 and U73122 (**C**), inhibition of Ca^2+^ entry with GSK7975a or Gd^3+^ (**G**), or both inhibitions with YM254890 and GSK7975a (**K**). Quantification of the area under the curve (AUC) of the Ca^2+^ responses in the control condition and treatments of the blockers (**D**,**H**,**L**). For panel (**H**), the AUC was calculated by omitting the first 3 min of the response that corresponded to the Ca^2+^ release phase, while for panels (**D**,**L**), the full responses were considered to calculate the AUC. Statistics were calculated with a Kruskal–Wallis test and Dunn’s multiple comparisons test (**D**,**H**) or with a Mann–Whitney test (**L**) **** *p* < 0.0001). Bars are mean ± SEM. For each condition, *n* varied between 307 and 731 cells from 7–16 independent experiments.

We then assessed the role of inositol-1,4,5-trisphosphate (IP_3_)-induced Ca^2+^ release by stimulating the cells in the presence of a PLC (phospholipase C) blocker (U73122) or a GPCRαq (G-protein-coupled receptor) inhibitor (YM254890) (Figure 4A–C). The inhibitors significantly decreased the fraction of RC that elicited a single Ca^2+^ transient, an oscillating pattern, or a sustained response, which was associated with an increase in nonresponding cells to around 60% (Figure 4C). In cells that still displayed an initial Ca^2+^ release, the amplitude was reduced by 45% (data not shown). Surprisingly, a significant proportion of cells (around 25%) did not respond with an initial Ca^2+^ elevation but started to display Ca^2+^ oscillations of rather large amplitude after 4–7 min (Figure 4B,C). To assess the overall impact on the Ca^2+^ response, we quantified the area under the curve (AUC), which was decreased by 79 and 72% after treatment with U73122 and YM254890, respectively (Figure 4D). From these experiments, we conclude that, as expected, IP_3_-induced Ca^2+^ release accounts for the first phase of the Ca^2+^ response and is responsible for most of the Ca^2+^ responses induced by serum.

Next, we tested the effect of Ca^2+^ entry inhibition using GSK7975a, an inhibitor of Orai1 and -2 channels [23], and the more general Ca^2+^ entry blocker Gd^3+^ (Figure 4E,F). We evaluated their effect by measuring the AUC of Ca^2+^ responses corresponding to Ca^2+^ entry, i.e., without considering the first 3 min that represent the Ca^2+^ release phase (see Figure 3A). This measure showed that the AUC was decreased by 66 and 49% in the presence of GSK7975a and Gd^3+^, respectively (Figure 4H). In addition, both inhibitors almost fully abolished the Ca^2+^ responses containing a sustained phase (Figure 4G). Finally, we combined both the inhibition of Ca^2+^ release and Ca^2+^ entry (YM254890 and GSK7975a). This led to a strong, but not full, inhibition of more than 90% of the Ca^2+^ response (evaluated by the AUC) to serum stimulation (Figure 4I–L).

### 3.3. Reserve Cell Activation Is Calcium Independent

**Figure 5:**
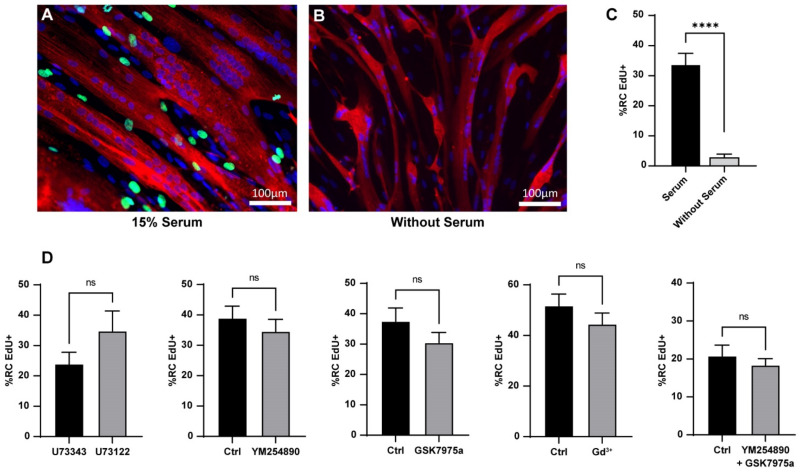
Heterogeneity of the serum-induced reserve cell activation and effect of Ca^2+^ signaling inhibitors. Myoblasts were differentiated for 48 h to obtain myotubes and RC, and EdU was added for 24 h to assess re-entry into the cell cycle of the RC. Immunofluorescence of the culture stimulated with 15% serum (**A**) or without serum (**B**); EdU in green, α-actinin in red; DAPI in blue. (**C**) Quantification of EdU + RC after 24 h of activation in the presence or not of serum. Statistics were measured with a Mann–Whitney test (**** *p* < 0.0001). Bars are mean ± SEM. For each condition, 27 images were quantified from three independent experiments. (**D**) Quantification of EdU + RC after 24 h of activation. The different inhibitors were added 10 min before serum stimulation and were present for the next 24 h. Statistics were calculated with Mann–Whitney tests. Bars are mean ± SEM. For each condition, 16–35 images were quantified from 2–6 independent experiments. ns: not significant.

Like the in vivo MuSC, RC are quiescent cells able to be activated after adequate stimulation [11,12]. We followed their re-entry into the cell cycle as a marker of their activation. To this end, we performed EdU staining for 24 h of stimulation with serum. Our data showed that 33.9 ± 3.5% of the RC exited the quiescent state and entered the cell cycle during the first 24 h of stimulation. This effect was due to the serum as the RC maintained in a medium without serum did not activate (3.1 ± 0.8%; Figure 5A–C).

To determine whether there is a link between the Ca^2+^ response and re-entry into the cell cycle, we induced RC activation in conditions where the IP_3_ pathway was blocked. To our surprise, neither U73122 nor YM254890 affected the percentage of RC that re-entered the cell cycle. In addition, Gd^3+^ or GSK7975a, which reduced Ca^2+^ entry, did not prevent the re-entry of RC into the cell cycle. Even the combination of YM254890 and GSK7975a did not have an impact on the activation of RC (Figure 5D).

**Figure 6:**
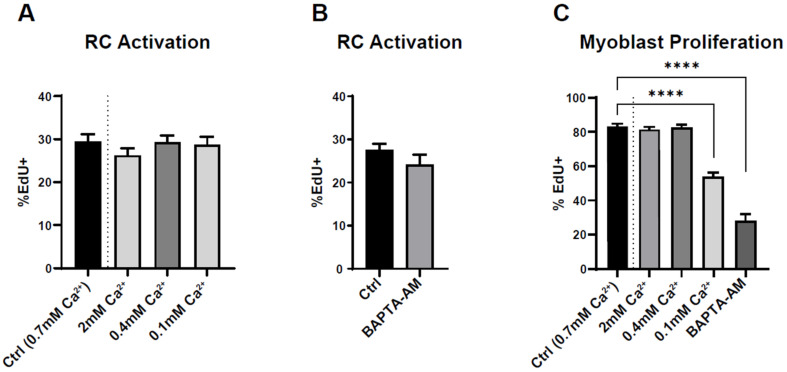
Effect of extracellular and intracellular Ca^2+^ concentration on reserve cell activation and myoblast proliferation. (**A**,**B**) Myoblasts were differentiated for 48 h to obtain myotubes and RC, followed by stimulation with 15% serum for 24 h in the presence of EdU. The extracellular Ca^2+^ concentration was modified 15 min before serum stimulation and kept for the next 24 h (**A**). Here, 10 µM BAPTA-AM was added to the medium 15 min before serum stimulation (**B**). Statistics for (**A**) and (**C**) were calculated with a Kruskal–Wallis test and Dunn’s multiple comparisons test relative to control (**** *p* < 0.0001), and statistics for (**B**) were measured with a Mann–Whitney test. Bars are mean ± SEM. A total of 40–71 images were quantified from 4–5 independent experiments.

These results strongly suggest Ca^2+^ independency of RC activation. To confirm this finding, we induced RC activation in the presence of various intra- or extracellular Ca^2+^ concentrations. We either increased the concentration of extracellular Ca^2+^ in the medium or decreased it by adding EGTA. Due to cell detachment, we could not use external Ca^2+^ concentration below 100 µM. We also incubated the cells with BAPTA-AM (10 µM), which prevented intracellular Ca^2+^ signals by around 80% (data not shown). None of these maneuvers impacted the ability of RC to activate upon serum addition (Figure 6A,B). As a control, we applied the same conditions for proliferating myoblasts maintained for several days in proliferating medium containing 15% serum. As expected, the proliferation of myoblasts was decreased when the intra- or extracellular Ca^2+^ concentration was significantly lowered (Figure 6C), showing the Ca^2+^ dependency of myoblast proliferation.

Finally, we stimulated RC with endothelin-1 (a GPCRαq activator). Endothelin-1 receptor B (EDNRB) is expressed almost exclusively in the human reserve cells compared to myotubes and proliferating myoblasts (RNA seq, data not shown). This treatment induced a robust Ca^2+^ signal in RC with a similar pattern of Ca^2+^ responses as serum stimulation, thereby confirming the inducing role exerted on calcium responses by IP_3_ pathway activation (Figure S2A,B). However, stimulation with endothelin-1 (100 nM) did not trigger RC activation (Figure S2C), showing that Ca^2+^ signals per se are not sufficient to activate RC.

### 3.4. Reserve Cell Migration Depends on Calcium Signals

**Figure 7:**
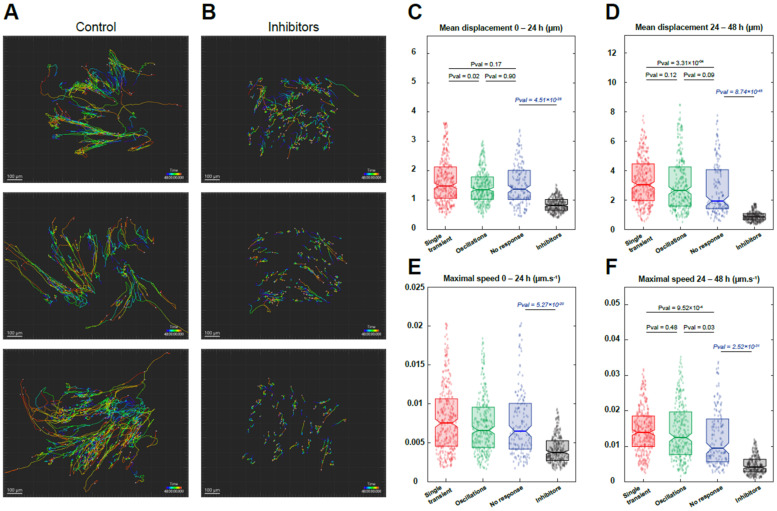
Migration of reserve cells. Myoblasts were differentiated for 48 h to obtain myotubes and RC. Cells were loaded with Cal520 to evaluate their Ca^2+^ response induced by serum and stained with SPY650-DNA to label the nuclei (see Materials and Methods for details). Ca^2+^ responses were recorded for 10 min, followed by timelapse imaging of 48 h to track the migration (**A**). For comparison, stimulation with YM254890 and GSK7975a (inhibitors) was performed (**B**). Mean displacement (distance between two time points separated by 15 min) of cells at 0–24 h (**C**) and 24–48 h (**D**), according to their Ca^2+^ response and compared with the condition in the presence of inhibitors. Maximum speed of cells at 0–24 h (**E**) and 24–48 h (**F**) depending on their Ca^2+^ response and compared with the condition in the presence of inhibitors. Statistics for “no response”, “single transient”, and “oscillations” were calculated with a Kruskal–Wallis test and multiple comparisons. Inhibitors were compared to “no response” with a Mann–Whitney test. The movement of 300–877 cells was followed from one (condition with inhibitors) to three independent experiments.

One of the early steps following MuSC activation in vivo is their migration to the place of injury [24,25]. After assessing the Ca^2+^ independency of RC activation, we investigated whether their migration potential upon serum activation was dependent on their patterns of Ca^2+^ responses. To this end, we first recorded the Ca^2+^ responses induced by serum addition before following the same cells for 48 h to evaluate their movement (Video S1). To limit the phototoxicity, Ca^2+^ recordings were carried out for only 10 min and at a low acquisition frequency (see Materials and Methods). This protocol enabled us to image the RC migration for 48 h without increasing the mortality of the cells but with a less precise Ca^2+^ response classification, thereby allowing us to only categorize the RC response as “no response”, “single Ca^2+^ transient”, and “oscillating pattern”. Cells were imaged for 48 h using phase contrast to differentiate the myotubes from the RC and using SPY650 staining to label the nuclei and track the cells (Video S2). We were thus able to follow the mean cell movement and the speed of each RC for 48 h and correlate it with their initial pattern of Ca^2+^ response (Figure 7). As shown in Figure 7, all RC displayed an increased distance and speed during the second part of the recording (24–48 h) compared to the first 24 h, independently of their Ca^2+^ responses, which suggests a period of latency of the cells upon stimulation. During the first 24 h, almost no difference was observed between the three categories of Ca^2+^ responses, except for a slight increase in the cell movement of those displaying a single Ca^2+^ transient (Figure 7C,D). After 24 h of recording, RC that exhibited Ca^2+^ response upon stimulation presented an increased mean distance compared to RC displaying no Ca^2+^ response (“no response” vs. “single Ca^2+^ transient” *p*-value = 3.31 × 10^−04^ and “no response” vs. “oscillations” *p*-value = 0.09). Accordingly, an increase in the maximum speed was also observed in RC with Ca^2+^ response (“no response” vs. “single Ca^2+^ transient” *p*-value = 9.52 × 10^−04^ and “no response” vs. “oscillations” *p*-value = 0.03) (Figure 7E,F). This result suggests that RC with a single Ca^2+^ transient are more mobile with a higher capacity to migrate compared to cells presenting other Ca^2+^ patterns. In parallel, cells with an oscillating Ca^2+^ pattern are less active in the first hours following serum stimulation but accelerate afterward. Finally, cells that do not display any Ca^2+^ response, while accelerating over time, remain slower and with reduced movement compared to other RC.

Following this characterization, we applied a combination of YM254890 and GSK7975a, the condition that prevented most of the Ca^2+^ signals (Figure 4L). We compared the cell migration in this condition to the data obtained by the cells displaying “no response” in the control as they were less mobile (Video S3). Upon stimulation in the presence of the inhibitors, migration of RC was markedly decreased in terms of both the mean distance and the maximum speed at any time of the recording (Figure 7), showing that calcium signals are essential for the migration of RC cells after their activation.

## 4. Discussion

This study identified RC responses following serum stimulation as heterogeneous through their capacity to re-enter the cell cycle, their Ca^2+^ responses upon stimulation, and their migration capacity. We showed that activation of RC was mainly independent of the Ca^2+^ signaling, while their capacity to migrate strongly relied on Ca^2+^ signals. Furthermore, the pattern of cytosolic Ca^2+^ responses affected the migration efficiency during the 48 h following serum stimulation.

The Ca^2+^ responses elicited by serum stimulation were heterogeneous among the RC population and could be categorized in five different patterns. We cannot rule out that a small part of the heterogeneity comes from cells that are not RC but already differentiated (while not fused) cells. According to a previous study [26] and our observation (data not shown), these cells committed to differentiation correspond to 10% or less of the mononucleated cell population. The different Ca^2+^ patterns were consistently observed between the experiments, while their proportion slightly varied between donors (the primary cells were from different donors but from the same muscle, see Materials and Methods). Around 90% of the cells responded with an initial Ca^2+^ elevation due to IP_3_R-induced Ca^2+^ release. This was followed or not by Ca^2+^ oscillations associated with a phase of sustained Ca^2+^ elevation in some cells. The second phase of the response was absent in experiments performed in a Ca^2+^-free medium, confirming the necessity of Ca^2+^ entry to maintain Ca^2+^ oscillations [27,28]. One possible route of Ca^2+^ entry is through the SOCE pathway. Indeed, the SOCE blocker GSK7975a [29] and Gd^3+^, a more general Ca^2+^ influx blocker, suppressed the plateau phase responses, and the AUC of the response was decreased by around 60% overall. However, surprisingly, neither GSK7975a nor Gd^3+^ prevented Ca^2+^ oscillations from taking place. Hence, it appears that Ca^2+^ entry occurs in part through the SOCE pathway but also through another route that remains to be determined. Potential candidates are the arachidonic-acid-regulated Ca^2+^-selective (ARC) channels [27] and TRP channels, with some being activated by second messengers such as diacylglycerol (produced together with IP_3_), even though one would expect these channels to be blocked by Gd^3+^. Alternatively, it could be that only a very limited entry of Ca^2+^ (not prevented by the blockers we used) was sufficient to drive the oscillations. When both SOCE and Ca^2+^ release were inhibited, it resulted in a strong reduction in the Ca^2+^ response to serum, with a decrease in AUC of more than 90%. However, a peculiar response consisting of the absence of initial Ca^2+^ elevation but followed by delayed oscillations was more frequently observed after blocking IP_3_ production. We did not investigate this type of Ca^2+^ pattern further, but it might be due to the production of other second messengers, such as nicotinic acid adenine dinucleotide phosphate (NAADP), acting on acidic compartments [30,31]. The complexity of this Ca^2+^ response is likely the consequence of using cell stimulation by serum, which contains hundreds of proteins that activate multiple pathways, with some resulting in Ca^2+^ signaling. Indeed, the Ca^2+^ response stimulated by endothelin-1 was fully abolished by blocking Ca^2+^ release and entry, without the appearance of delayed oscillations (data not shown). Hence, endothelin-1 triggers a “simpler” response compared to serum and strongly suggests that at least two intracellular signaling pathways are activated by serum, thereby leading to cytosolic Ca^2+^ elevation. While this complicates a comprehensive analysis of Ca^2+^ response, stimulation by serum better mimics the physiological situation where multiple factors activate MuSC concomitantly [32].

In the control condition, serum stimulation promoted re-entry into the cell cycle of around 30% of RC measured after 24 h. Unexpectedly, none of the procedures that limited the Ca^2+^ response negatively impacted RC activation, even those that almost entirely prevented cytosolic Ca^2+^ elevation, such as the combination of YM254890 and GSK7975a or the incubation of cells with BAPTA-AM. These results strongly indicate an RC activation process largely independent of Ca^2+^ signals. Another argument in favor of the Ca^2+^ independency of the RC activation process is the stimulation by rndothelin-1, which induced a robust Ca^2+^ response. Even though the Ca^2+^ responses were similar to the one induced by serum, no activation of RC was induced, leading us to conclude that a rise in cytosolic Ca^2+^ concentration per se is insufficient to promote cell activation. These results imply that RC activation depends on other stimuli present in serum in addition to those inducing the Ca^2+^ response. In contrast, the proliferation of myoblasts was decreased when cytosolic Ca^2+^ fluctuations were prevented by the presence of BAPTA-AM or when a low extracellular Ca^2+^ medium was used, showing that cell proliferation is a Ca^2+^-dependent process, as reported on many different cellular systems [33,34,35,36]. Somatic stem cells share a certain degree of similarity with cancer stem cells [37]. It is thus of interest to note that the ability of RC to re-enter the cell cycle (as shown by the incorporation of EdU) in a manner independent of Ca^2+^ signals is reminiscent of findings obtained in the 1970s showing that DNA synthesis of cancer cells (in contrast to nontumoral cells) was largely insensitive to Ca^2+^ concentration and that cancer cells were able to grow in very low extracellular Ca^2+^ environment [38,39,40]. More recently, the group of Capiod also reported cell proliferation occurring in the absence of external Ca^2+^ and pointed to a role of Ca^2+^ channels, but not necessarily Ca^2+^ entry, as being important for cell proliferation, at least in some cellular systems [41]. However, other studies have highlighted the importance of Ca^2+^ entry, and in particular SOCE, for cancer cell proliferation [42,43]. From our data, we propose that the activation of RC, meaning the exit from the G_0_ phase and the initial DNA synthesis, takes place regardless of the Ca^2+^ concentration. Once this first step is achieved, the myoblasts then require the generation of Ca^2+^ signals for their proliferation. Thus, our in vitro approach using quiescent RC allows clear differentiation between activation and proliferation.

Very few studies have investigated the Ca^2+^ response of MuSC after stimulation. One study recorded the Ca^2+^ response of MuSC still attached to freshly isolated FDB (flexor digitorum brevis) fibers with a very low sampling rate (one point every 5 min) [21]. Cell stimulation with FGF2 induced a very slow Ca^2+^ elevation that was prevented by the TRPC channel blocker SK&F 96365. This Ca^2+^ signal was associated with the transcription factor MyoD expression and the translocation of NFAT1 (nuclear factor of activated T-cells 1) and NFAT4 into the nucleus. Both events were blocked by SK&F 96365, leading to the conclusion that satellite cell activation by FGF2 (fibroblast growth factor 2) is a process dependent on Ca^2+^ signaling [21]. Another study reported the stretch-induced Ca^2+^ elevation of isolated satellite cells. Ca^2+^ elevation was due to the sequential activation of stretch-activated cation channels (blocked by GsMTx-4) followed by voltage-gated L-type Ca^2+^ channel opening (blocked by nifedipine). Increase in Ca^2+^ eventually stimulated NO (nitric oxide) production, which in turn led to MMP2 (matrix metalloproteinase-2) production by MuSC that releases HGF (hepatocyte growth factor) from the extracellular matrix. Finally, by binding to its receptor c-met, HGF activated MuSC [20]. Interestingly, while the cellular NO production depended on stretch-induced Ca^2+^ entry, stimulation of the cells directly with HGF promoted MuSC activation independently of Ca^2+^ entry (see Figure 2 from Hara et al. 2012). Hence, the stretch-induced release of the growth factor was Ca^2+^ dependent, while direct activation of MuSC by HGF occurred independently of Ca^2+^ entry.

MuSC activation is accompanied by cell migration in the repair site, an essential step toward muscle regeneration [44]. Migration involves many steps that occur both at the front and rear of migrating cells. Cell extension at the front (lamellipodia) is supported by actin polymerization and anchoring at the nascent focal adhesion site. At the rear, mature focal adhesions need to be destabilized for the cell to move forward. Focal adhesion dynamics are Ca^2+^ dependent, with Ca^2+^-dependent protease calpain notably acting at the rear of the cells, inducing proteolysis of talin [45] and FAK (focal adhesion kinase) [46]. It has been known for several decades that a cytosolic Ca^2+^ gradient from rear to front is established in migrating cells [47]. Ca^2+^ release events occur at the front [48], while SOCE has been shown to be more dominant at the rear of cells [49]. We analyzed the migratory behavior of RC upon serum stimulation, and our results showed that strong inhibition of the Ca^2+^ response with YM254890 and GSK7975a had a significant impact on the RC mobility, with both the speed and distance covered by the RC reduced during the 48 h of analysis. We measured the Ca^2+^ response of each RC for 10 min and followed the exact same cells for the next 48 h to determine a possible correlation between the pattern of Ca^2+^ responses and the migration capacity. We separated the cells into three categories, namely nonresponders, those displaying only a single Ca^2+^ transient, and those having an oscillatory response. In line with reduced movement in the presence of Ca^2+^ blockers, the nonresponding cells moved less and slower than the responding ones. Cells exhibiting only a single Ca^2+^ transient displayed a significantly higher movement during the initial 24 h compared to those showing Ca^2+^ oscillations. However, no significant difference was observed between these two populations in the last 24 h of recording. To note, even the cells that did not display a cytosolic Ca^2+^ elevation during the first 10 min of serum stimulation presented a higher migration capacity than cells treated with Ca^2+^ blockers. This could be explained by the almost complete prevention of any Ca^2+^ signals for 48 h in the presence of blockers, while the cells classified as nonresponders might have generated Ca^2+^ signals after the initial 10 min of the recording. Interestingly, irrespective of the type of Ca^2+^ response (even no response), the cells moved less overall during the initial 24 h of serum stimulation, with the movement accelerating after 24 h.

In conclusion, we have provided, for the first time to our knowledge, detailed analysis of the Ca^2+^ responses elicited by in vitro MuSC upon serum stimulation. These responses displayed very heterogeneous patterns that were surprisingly not correlated to the ability of RC to exit the G_0_ phase and enter the cell cycle. In contrast, the pattern of Ca^2+^ responses influenced the migratory potential of these cells. Hence, we have revealed that the two important steps of muscle regeneration, namely the activation and migration of MuSC, differently rely on Ca^2+^ signals.

## Data Availability

The data presented in this study are openly available in Yareta https://yareta.unige.ch/#/home (at 10.26037/yareta:5ie4m42p7va7xkrk3vb5rvfjzy).

## Supplementary Materials

The following supporting information can be downloaded at: https://www.mdpi.com/article/10.3390/cells11101689/s1, Figure S1: ATP-induced Ca2+ responses in RC and myotubes; Figure S2: Stimulation with endothelin-1 triggers Ca2+ responses in RC but not their re-entry in the cell cycle; Video S1: Example of serum-induced calcium responses in RC; Video S2: Analysis of the 48 h RC migration in the control condition; Video S3: Analysis of the 48 h RC migration in YM254890 and GSK797.
