# Supplementary material for: Activation and Migration of Human Skeletal Muscle Stem Cells In Vitro Differently Rely on Calcium Signals
Source: Cells. 2022 May 19;11(10):1689. doi: 10.3390/cells11101689 (PMC9140175; doi:10.3390/cells11101689)
Supplement: Supplementary file 1 [file cells-11-01689-s001.zip › Legends Movies.pdf]

Movie 1: Example of serum-induced calcium responses in RC. Myoblasts were differentiated for 48 h to obtain myotubes and RC, and the culture was loaded with Cal520 and SPY650 before being stimulated with a 15% serum. To identify the calcium signature of each cell, cells were recorded for 10 min (1 picture every 15 sec).

Movie 2: Analysis of the 48 h RC migration in the control condition. The first frame represents the area used for the calcium imaging (objective 20X). The following recordings used the objective 10X at the same position for measuring the migration. Nuclei stained with SPY650 are represented in white (RC with an early identified calcium response induced by serum) or blue (mainly the nuclei from the myotubes or cells outside the calcium recording frame). Lines represent the migration tail of each cell with a color variation associated with the time.

Movie 3: Analysis of the 48 h RC migration in YM254890 and GSK7975a. The analysis parameters were the same as the one described for movie 2, and drugs were added 10 min before the serum stimulation and kept during recording.
