# Supplementary material for: Activation and Migration of Human Skeletal Muscle Stem Cells In Vitro Differently Rely on Calcium Signals
Source: Cells. 2022 May 19;11(10):1689. doi: 10.3390/cells11101689 (PMC9140175; doi:10.3390/cells11101689)
Supplement: Supplementary file 1 [file cells-11-01689-s001.zip › Tollance Figure Sup_RGB.pdf]

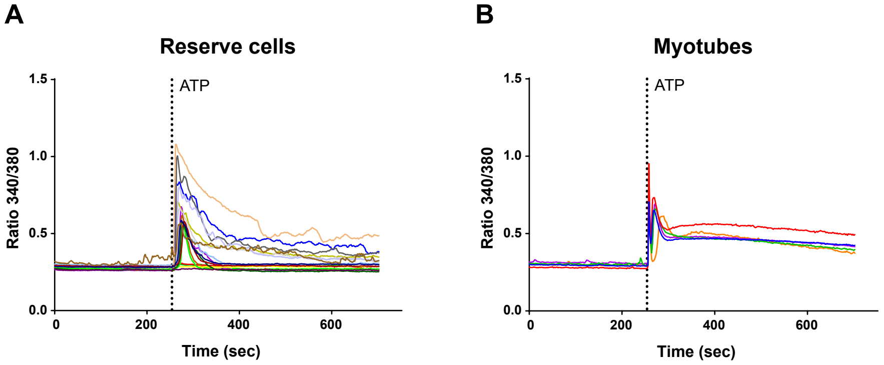

Figure S1: ATP-induced Ca^{2+} responses in RC and myotubes. Myoblasts were differentiated during 48 h to obtain myotubes and RC and the culture was loaded with Fura2 and stimulated with 100 µM of ATP. The response of RC (A) and myotubes (B) is shown. Each panel is the response of a representative coverslip.

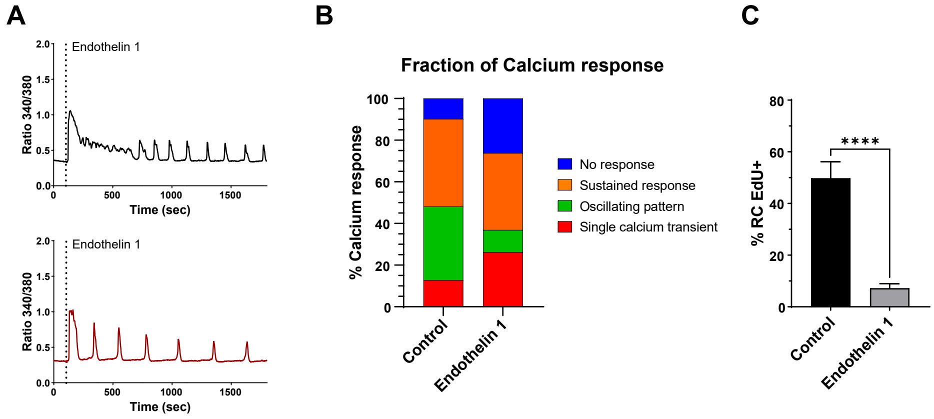

Figure S2: Stimulation with Endothelin 1 triggers Ca^{2+} responses in RC but not their re-entry in the cell cycle. Myoblasts were differentiated during 48 h to obtain myotubes and RC, loaded with Fura-2 and stimulated with 15% serum or Endothelin 1 (100 nM). (A) Representative examples of sustained responses and Ca^{2+} oscillations obtained with Endothelin 1 stimulation. (B) The percentage of each type of response is represented. (C) The same conditions were used to assess RC activation during 24 h with EdU. Statistics were done with a Mann-Whitney test (**** p < 0,0001). Bars are mean ± SEM, 10 images were quantified from 2 independent experiments.
